# Senescence of alveolar epithelial cells impacts initiation and chronic phases of murine fibrosing interstitial lung disease

**DOI:** 10.3389/fimmu.2022.935114

**Published:** 2022-08-18

**Authors:** Zento Yamada, Junko Nishio, Kaori Motomura, Satoshi Mizutani, Soichi Yamada, Tetuo Mikami, Toshihiro Nanki

**Affiliations:** ^1^ Department of Internal Medicine, Toho University Graduate School of Medicine, Tokyo, Japan; ^2^ Division of Rheumatology, Department of Internal Medicine, Toho University School of Medicine, Tokyo, Japan; ^3^ Department of Immunopathology and Immunoregulation, Toho University School of Medicine, Tokyo, Japan; ^4^ Department of Pathology, Toho University School of Medicine, Tokyo, Japan

**Keywords:** interstitial lung disease, p21, p16, senescence-associated secretary phenotype, type 2 alveolar epithelial cell, IL-6, interstitial macrophages

## Abstract

Fibrosing interstitial lung disease (ILD) develops due to the impaired reparative processes following lung tissue damage. Cellular senescence has been reported to contribute to the progression of fibrosis. However, the mechanisms by which these senescent cells initiate and/or drive the progression of lung tissue fibrosis are not yet fully understood. We demonstrated that p21^WAF1/CIP1^- and p16^INK4A^-pathway-dependent senescence in type 2 alveolar epithelial cells (AEC2) were both involved in the initiation and progression of lung fibrosis in murine bleomycin (BLM)-induced ILD. p21^WAF1/CIP1^-senescent AEC2 emerged rapidly, as early as 1 day after the intratracheal instillation of BLM. Their number subsequently increased and persisted until the later fibrosis phase. Very few p16^INK4A^-senescent AEC2 emerged upon the instillation of BLM, and their increase was slower and milder than that of p21^WAF1/CIP1+^ AEC2. AEC2 enriched with senescent cells sorted from BLM-ILD lungs expressed senescence-associated secretory phenotype (SASP)-related genes, including *Il6*, *Serpin1*, *Tnfa*, *Ccl2*, *Tgfb*, and *Pdgfa*, at the initiation and chronic phases of fibrosis, exhibiting distinct expression patterns of magnitude that were dependent on the disease phase. Ly6C^+^ inflammatory monocytes increased in the lungs immediately after the instillation of BLM and interstitial macrophages increased from day 3. The expression of *Acta2* and *Col1a1* was upregulated as early as day 1, indicating the activation of fibroblasts. We speculated that IL-6, plasminogen activator inhibitor-1 (PAI-1), and TGF-β contributed to the accumulation of senescent cells during the progression of fibrosis in an autocrine and paracrine manner. In addition, CCL2, produced in large amounts by senescent AEC2, may have induced the infiltration of Ly6C^+^ inflammatory monocytes in the early phase, and TGF-β and PDGFa from senescent AEC2 may contribute to the activation of fibroblasts in the very early phases. Our study indicated that senescent AEC2 plays a role in the pathogenesis of fibrosing ILD throughout the course of the disease and provides insights into its pathogenesis, which may lead to the development of new therapeutic methods targeting senescent cells or SASP molecules.

## Introduction

Fibrosing interstitial lung diseases (ILDs) are a group of diseases in which excessive extracellular matrix proteins are produced by persistently activated fibroblasts causing the destruction of the alveolar architecture (1). Fibrosis occurs in the normal wound healing process and resolves over time. However, continuous inflammation and unresolved cellular damage impair reparative processes, leading to the progression of fibrosis and tissue remodeling (2). While smoking (3) and the inhalation of silica (4) are well-known environmental factors that cause idiopathic pulmonary fibrosis (IPF), several genetic variants for surfactant protein-C, surfactant protein-A2, telomerase reverse transcriptase, telomerase RNA component, and MUC5B have been identified as factors associated with IPF or familial interstitial pulmonary fibrosis (5–8). Autoimmune reactions are also triggering factors that initiate tissue injury in fibrosing ILD in association with connective tissue diseases, including rheumatoid arthritis, systemic scleroderma, polymyositis, and dermatomyositis (9). Regardless of the triggers and/or causative genetic variants, the molecular mechanisms underlying tissue/cellular damage and resultant fibrosis are not fully understood, and they involve complex interactions between immune cells, epithelial cells, fibroblasts, and endothelial cells (9).

The paradigm that the damage of alveolar epithelial cells (AECs) and impaired reparative processes drive lung fibrosis has recently become prominent in the pathogenesis of ILD (10). AECs are classified as type 1 (AEC1) and type 2 (AEC2). AEC2 are progenitor cells that self-renew and transdifferentiate into AEC1, and are also producers of surfactant phospholipids and proteins, which reduce surface tension at the alveolar air-liquid interface (10). Continuous environmental stimuli from airways have been suggested to cause AEC2 dysfunction, such as a hyper-activated state, apoptosis, or senescence, leading to inflammatory cell infiltration and parenchymal fibrosis in the lungs.

Cellular senescence is a state of permanent cell cycle arrest induced by various types of extrinsic or intrinsic stresses, including DNA damage by telomere shortening, genotoxic stress, reactive oxidative stress, mitochondrial dysfunction, and oncogene activation (11, 12). These stressors activate the p53-p21^WAF1/CIP1^ and/or the p16^INK4A^-pRB pathways. p21^WAF1/CIP1^ and p16^INK4A^ are both cyclin-dependent kinase (CDK) inhibitors that inhibit the kinase activity of cyclin–CDK complexes, which are required for cell cycle progression (13). The prolonged activation of either pathway, p53-p21^WAF1/CIP1^ or p16^INK4A^-pRB, is sufficient to induce senescence (13). Although p21^WAF1/CIP1^ is more relevant for the initiation of senescence, the expression of p16^INK4A^ is critical for persistent senescence (14).

Cell cycle markers, typically p21^WAF1/CIP1^, p16^INK4A^, p53, phospho-p53, a decrease in pRB, and the absence of proliferation, are used to detect cellular senescence. However, these are not universal markers because they are also expressed in non-senescent cells (15). Moreover, whereas p53-p21^WAF1/CIP1^ is transiently activated and more relevant for the initiation of senescence, the expression of p16^INK4A^ is critical for persistent senescence (14). Therefore, additional hallmarks are used in combination to conclusively identify senescence: structural changes associated with senescence, such as an increased lysosomal mass detected by a morphologically enlarged cell size; senescence-associated β-galactosidase (SA-βgal) activity; changes in organelle structures represented by the downregulation of Lamin B1; and nuclear alterations detected by DNA damage response-associated proteins or phosphorylated H2A histone family member X (γ-H2AX) (15).

Although cell growth is arrested, senescent cells remain metabolically active and acquire a senescence-associated secretory phenotype (SASP), by which senescent cells produce a wide array of soluble molecules, including cytokines, chemokines, matrix remodeling proteases, extracellular matrix components, and growth modulators (11, 13). These mediators alter the state of the surrounding cells. In addition, SASP promotes senescence in an autocrine manner and that of neighboring cells in a paracrine manner, thereby spreading senescent cells throughout the entire tissue (12, 13, 16).

Cellular senescence was recently reported to contribute to various age-related inflammatory diseases, including obesity, atherosclerosis, osteoarthritis, Alzheimer’s disease, and lung diseases, such as IPF (17). Since senescent cells are resistant to apoptosis, they accumulate in aged organs, leading to these inflammatory diseases due to SASP (17). SA-βgal, p16^INK4A^, p53, or p21^WAF1/CIP1^ and disease-dependent SASP are present at inflamed sites. In murine disease models, these diseases are ameliorated by the elimination of senescent cells using genetically engineered mice or senolytics (18–21).

Previous studies have shown that in human IPF, DNA damage and expressions of SA-βgal, p16^INK4A^, p53, or p21^WAF1/CIP1^ have been observed in lung fibroblasts and/or epithelial cells (22–28). Similar to human IPF, senescence markers or SASP have been detected in AEC and/or fibroblasts in bleomycin-induced ILD (BLM-ILD) (29–33). Since BLM elicits DNA damage in cell lines and primary cells, including epithelial cells and fibroblasts (34), its intratracheal instillation is considered to induce DNA damage and resultant senescence in the lung cells of BLM-ILD. However, these studies have stressed the importance of the senescence of different cell types, including AECs, fibroblasts, and endothelial cells, in BLM-ILD.

Although the emergence of senescent cells has been demonstrated in human IPF as well as in its murine model, the mechanisms by which these senescent cells initiate and/or promote the progression of lung tissue fibrosis are not yet fully understood. Furthermore, most studies on murine BLM-ILD have analyzed SASP-associated mediators in the chronic phase of lung fibrosis on day 14 or 28 after the instillation of BLM. No studies have focused on the role of senescence at the initiation phase. Furthermore, the link between SASP and pathogenesis, such as immune cell infiltration and fibroblast activation, remains unknown.

To investigate how cellular senescence and SASP contribute to the development and progression of fibrosing ILD, we investigated the dynamics of p21^WAF1/CIP1^- and p16^INK4A^-dependent senescence from disease initiation to the established fibrosis phase in murine BLM-ILD. We found that type 2 AEC (AEC2) expressed both p21^WAF1/CIP1^ and p16^INK4A^ proteins upon the BLM instillation with a distinct pattern of expression dynamics. Senescent cell-enriched AEC2 subsets highly expressed a set of inflammatory SASP-related genes at both the initiation and fibrosis phases. These inflammatory mediators may promote the infiltration of monocytes and activation of fibroblasts from the early phase of the progression of fibrosis. These results suggest a critical role for senescent AEC2 throughout the course of fibrosing ILD and provide insights into its pathogenesis, which may lead to the development of new therapeutic methods targeting senescent cells or SASP molecules.

## Results

### p21^WAF1/CIP1^-expressing cells in the early phase in BLM-ILD

**Figure 1:**
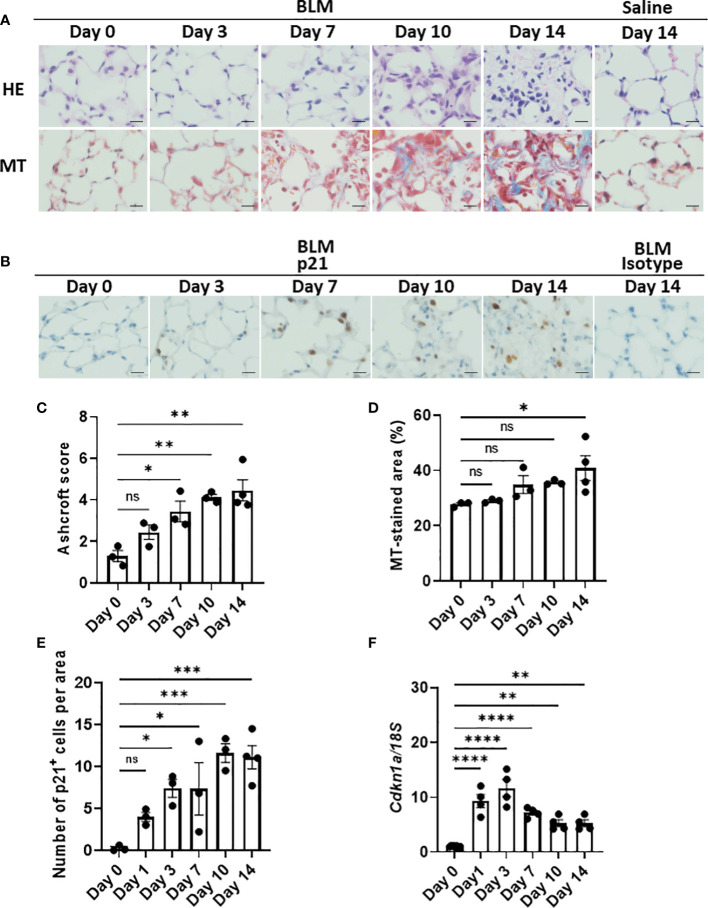
Emergence of p21WAF1/CIP1+ cells in lungs in the early phase of bleomycin-induced ILD (BLM-ILD).C57BL/6 mice were intratracheally instilled with BLM to induce BLM-ILD. Control mice were intratracheally instilled with saline. The lungs were obtained before and 1, 3, 7, 10, and 14 days after the instillation of BLM and 14 days after the instillation of saline. **(A)** Representative images of hematoxylin and eosin (HE) and Masson’s trichrome (MT) staining of paraffin-embedded lung tissue on the indicated days. **(B)** Representative images of the immunohistochemical staining of p21WAF1/CIP1 of lung tissue from mice on the indicated days. **(C)** The Ashcroft scale using MT-stained lung tissue from pooled mice. **(D)** Percentage of the collagen fiber area in MT-stained lung tissue. The MT-stained area was measured by ImageJ in 20 randomly selected areas (360 × 240 µm) and the average was plotted. **(E)** Number of p21WAF1/CIP1+ cells per area of the right lung from pooled mice. p21WAF1/CIP1+ cells were counted in 10 randomly selected areas (180 × 120 µm) and the average was plotted. **(F)** Cdkn1a expression relative to 18S rRNA (18S) of the lung by quantitative RT-PCR (qRT-PCR). Scale bars indicate 5 µm. n = 3–4 per day **(C–E)**; n = 4 per day **(F)**. Data are presented as the mean ± SEM. Statistics show p-values from a one-way ANOVA with Dunnett’s multiple comparisons test as a post-hoc test comparing values on day 0. ns, not significant. *p < 0.05; **p < 0.01; ***p < 0.001; ****p < 0.0001.

Although the emergence of senescent cells in BLM-ILD has been demonstrated, most studies detected these cells in the late phase of fibrosis. To elucidate the dynamics of the emergence of senescent cells, we first examined the expression of p21^WAF1/CIP1^, a cell cycle arrest marker, in lung tissue by immunohistochemistry in parallel with an evaluation of histological fibrosis during the progression of BLM-ILD. Lung tissue stained with hematoxylin and eosin (HE) and Masson’s trichrome (MT) exhibited normal alveolar wall thickness and very limited infiltration in a small area at day 3 post-BLM instillation (**Figure 1A**). The infiltration and thickening of alveolar septa appeared on day 7. The accumulation of collagen became evident and gradually increased after day 7. The extensive formation of fibrotic loci was observed after day 10. Mice intratracheally instilled with saline did not show any cell infiltration or septal collagen fibers, even on day 14 (**Figure 1A**). The Ashcroft score (35), a semi-quantitative method to score lung fibrosis based on cell infiltration and the accumulation of collagen, showed that the area of cell infiltration with accumulated collagen gradually increased (**Figure 1C**). The quantitative analysis of MT-stained sections revealed a gradual increase in collagen deposition from the early phase to the fibrosis phase on day 14 (**Figure 1D**). Cells expressing the senescence marker p21^WAF1/CIP1^ appeared in some alveoli and were scattered throughout the lungs on day 3 post-BLM instillation, when fibrosis was not evident by HE or MT staining (**Figure 1B**). The number of p21^WAF1/CIP1+^ cells increased after day 3 and were more concentrated in fibrotic loci on days 10 and 14. Most p21^WAF1/CIP1+^ cells were morphologically enlarged, which is a characteristic of senescent cells. The quantitative analysis revealed that the number of p21^WAF1/CIP1+^ cells was increased as early as on day 1. After day 3, the number of p21^WAF1/CIP1+^ cells was significantly higher than on day 0, and gradually increased, persisting after day 10 (**Figure 1E**). In contrast, the expression of *Cdkn1a*, encoding p21^WAF1/CIP1^, was strongly induced as early as day 1 after the instillation of BLM. However, it decreased from day 7 (**Figure 1F**). The divergence of expression dynamics between p21^WAF1/CIP1^ and *Cdkn1a* might be attributed to the transient expression of p21^WAF1/CIP1^ in some cells upon the BLM stimulation and the post-translational regulation of p21^WAF1/CIP1^ (see *Discussion*). Thus, these results suggested that the cellular senescence of alveolar cells was induced immediately upon the BLM instillation and preceded the histological formation of fibrosis.

**Figure 2:**
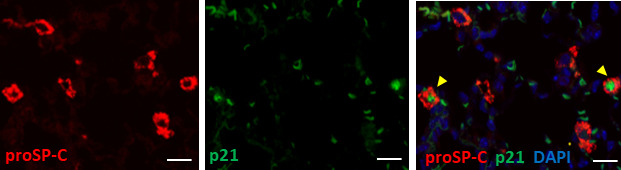
Type 2 alveolar epithelial cells (AEC2) undergo senescence in BLM-ILD.Paraffin-embedded lung tissue sections from mice 14 days after the instillation of BLM were used for double immunofluorescence staining for prosurfactant protein C (proSP-C) (red) and p21WAF1/CIP1 (p21) (green) with 4,6-diamidino-2-phenylindole (DAPI) (blue) counterstaining for nuclei. Yellow arrows indicate double-positive cells. The scale bar indicates 20 µm.

We speculated that most p21^WAF1/CIP1+^ cells were AEC2 based on their spherical shape and relatively large round nuclei, as shown in **Figure 1B**. As expected, double color immunofluorescence of p21^WAF1/CIP1^ and prosurfactant protein C (proSP-C), a marker of AEC2, showed that p21^WAF1/CIP1^ was stained on the nuclei of proSP-C-positive AEC2 (**Figure 2**). Approximately 30% of AEC2 were positive for p21 ^WAF1/CIP1^ (**Supplementary Table 1**). Taken together, these results indicated that AEC2 highly expressed p21^WAF1/CIP1^ from the initiation phase of BLM-ILD and that p21^WAF1/CIP1+^ AEC2 increased and persisted until the later fibrosis phase.

### Distinct expression dynamics of p16^INK4A^ in the progression of lung fibrosis in BLM-ILD

**Figure 3:**
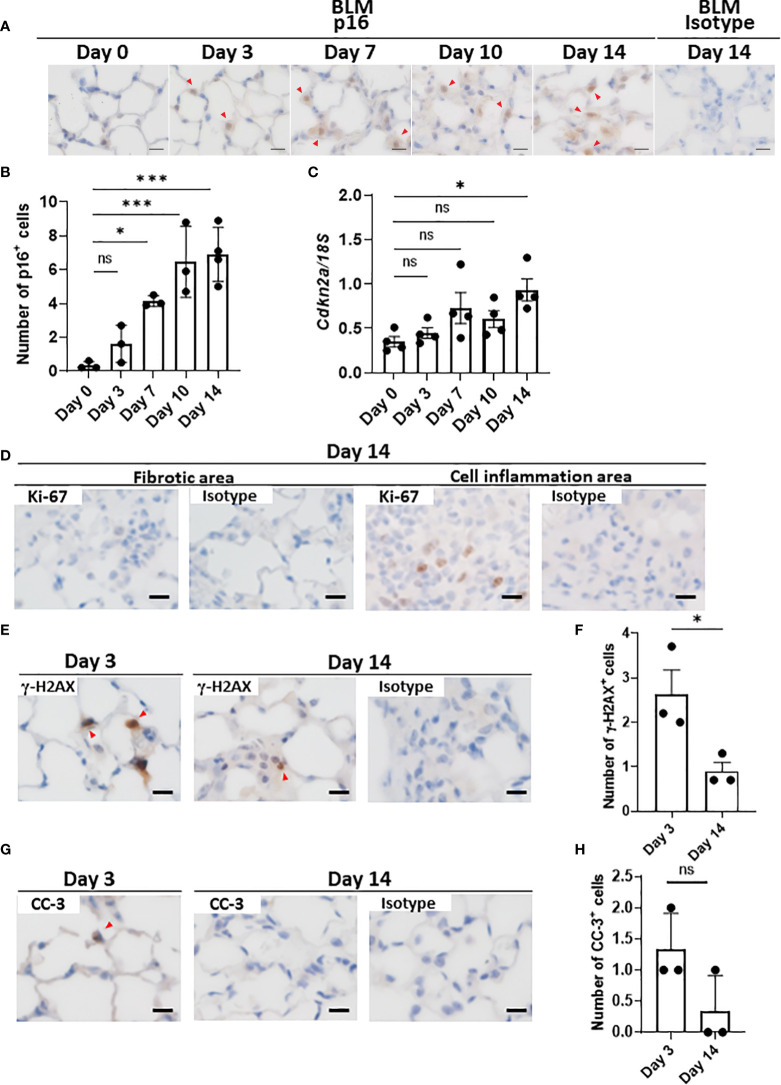
AEC2 in BLM-ILD express other cellular senescence markers.Paraffin-embedded lung tissue was prepared as described in **Figure 1**. **(A)** Representative images of immunohistochemical staining for p16INK4A on the indicated days. **(B)** Numbers of p16INK4A+ cells per area of the right lung from pooled mice. p16INK4A+ cells were counted in 10 randomly selected areas (180 × 120 µm) and the average was plotted. n = 3–4 per day. **(C)** Cdkn2a expression relative to 18S rRNA in the lungs of pooled mice by qRT-PCR. n = 4 per day. **(D–H)** Immunohistochemical analysis of Ki-67 **(D)**, γ-H2AX **(E, F)**, and cleaved caspase-3 (CC-3, **G, H**) in the lungs of mice 3 and/or 14 days after the instillation of BLM. The numbers of γ-H2AX+ **(F)** cells per area or the total number of CC-3+ **(H)** cells in 10 areas were plotted as described in **Figure 1E**. Red arrows indicate positive cells. Scale bars indicate 5 µm. Data are presented as the mean ± SEM. A one-way ANOVA with Dunnett’s multiple comparisons test was used as a post-hoc test comparing values on day 0 **(B, C)**. Unpaired t-tests were used for two-group comparisons **(F, H)**. ns, not significant. *p < 0.05; ***p < 0.001.

Since there are no highly sensitive and specific markers of cellular senescence (15, 36), we examined the expression of another cell cycle arrest marker, p16^INK4A^, in the lungs throughout the progression of BLM-ILD. We observed the expression of p16^INK4A^ in the nuclei of a very small number of AEC as early as on day 1, and p16^INK4A+^ cells increased as the disease progressed (**Figures 3A, B**), but were not detected on day 0. Most p16^INK4A+^ cells had a spherical shape, suggesting AEC2. They were also enlarged, similar to p21^WAF1/CIP1+^ cells. It is important to note that increases in the number of p16^INK4A+^ AEC2 were milder and slower in the early phase (**Figure 3B**) than those in p21^WAF1/CIP1+^ AEC2 (**Figure 1E**). p16^INK4A+^ AEC2 exhibited a marked increase after day 7 (**Figure 3B**). The expression of *Cdkn2a* encoding p16^INK4A^ gradually increased upon the BLM instillation, with a significant increase being observed on day 14 (**Figure 3C**). These results showed that the slight increase in p16^INK4A+^ AEC2 in the early phase was in contrast to the sudden emergence of a relatively large number of p21^WAF1/CIP1+^ AEC2 upon the BLM instillation. Moreover, p16^INK4A^ and p21^WAF1/CIP1^ were both persistently expressed in AEC2 in the later fibrosis phase, indicating that senescence dependent on both pathways continued to exist in AEC2 of fibrotic lung tissue. Therefore, p16^INK4A^ expression may be more strongly affected by tissue environmental changes induced after the BLM instillation rather than by the direct effects of BLM.

### AEC2 in BLM-ILD lungs show senescent cell characteristics

As described above, a combination of markers is used to prove the cellular senescence of AEC2. Although SA-βgal is a common marker for identifying senescent cells, it was not possible to measure its enzyme activity in the paraffinized sections used in this study. AEC2 did not express Ki-67 in fibrotic areas in the later phases (**Figure 3D**), supporting the lack of proliferation, which is also a characteristic of senescence. In contrast, Ki-67^+^ cells from the same lung section were localized in the area with cell infiltration, in which senescent cells were absent (**Figure 3D**). We also examined γ-H2AX, indicating DNA damage, which is another hallmark of senescent cells, in BLM-ILD on day 3 (**Figure 3E**). We found that γ-H2AX-stained cells were morphologically identified as enlarged AEC2, indicating that double-stranded DNA damage was induced in these cells by BLM (**Figures 3E, F**). On the other hand, its expression decreased on day 14 (**Figures 3E, F**). Since DNA damage may lead to apoptosis, we also examined cleaved caspase 3 (CC-3). Although a subset of cells with the shape of AEC2 expressed CC-3 on day 3, very few cells expressed it on day 14 (**Figures 3G, H**). These results suggested that the majority of p21^WAF1/CIP1^-expressing AEC2 cells had acquired senescence, but that a small subset of p21^WAF1/CIP1+^ AEC2 cells underwent apoptosis due to BLM-induced double-stranded DNA breaks in the early phase of BLM-ILD. These results also indicated that the persistent expression of p21^WAF1/CIP1^ in the later fibrosis phase was not driven by DNA damage. Collectively, enlarged AEC2 were positive with another cell cycle arrest marker, p16^INK4A^, and did not proliferate, indicating that a subset of AEC2 were senescent cells. Furthermore, the senescence of AEC2 occurred due to DNA breakage through the direct effects of BLM in the early phase; however, persistent senescence in the fibrosis phase may be driven by other factors. This senescence appeared to be dependent on environmental alterations caused by tissue damage.

### AEC2 acquired SASP in BLM-ILD

**Figure 4:**
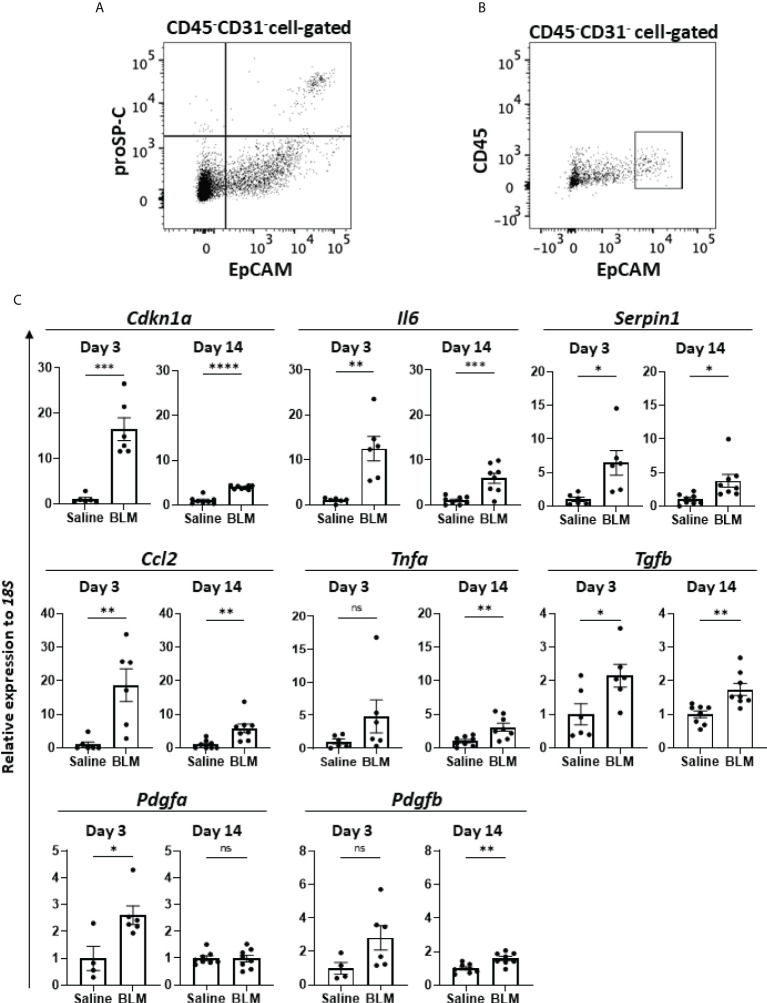
SASP-acquired AEC2 express multiple inflammatory mediators in BLM-ILD lungs. A cell suspension was obtained from the lungs of mice 14 days after the instillation of BLM and stained with fluorochrome-conjugated antibodies for a flow cytometric analysis. **(A)** After surface staining with monoclonal antibodies (mAbs) for CD31, CD45, and EpCAM, intracellular staining with an anti-proSP-C polyclonal antibody and Alexa-Fluor 555-conjugated secondary antibody was performed. **(B)** Gating of the CD45-CD31-EpCAMhi cell population sorted as AEC2. **(C)** Relative expression of the indicated SASP-related genes in sorted CD45-CD31-EpCAMhi cells from BLM-ILD lungs 3 and 14 days after the instillation of BLM. n = 6 on day 3; n = 8 on day 14. Data are presented as the mean ± SEM. Statistics show p-values from the unpaired t-test. ns, not significant. *p < 0.05; **p < 0.01; ***p < 0.001; ****p < 0.0001.

Previous studies reported the senescence of fibroblasts, AEC, and/or endothelial cells in human IPF (22–28) as well as in murine BLM-ILD (29–33). However, which senescent cells exhibit the SASP-mediated production of inflammatory mediators and contribute to the development of lung fibrosis has not been clarified. Since we found a robust senescence phenotype in AEC2 from the early phase to the fibrosis phase, we hypothesized a critical role for the cellular senescence of AEC2 in fibrosing ILD. Therefore, we examined the gene expression of SASP-related mediators of AEC2 in the early phase before the accumulation of collagen as well as in the later phases of fibrosis, and investigated whether senescent AEC2 acquire SASP and contributes to the initiation/progression of fibrosing ILD. It was challenging to collect live AEC2 because of the limited availability of surface markers. We confirmed that proSP-C, an intracellular marker of AEC2, was expressed in the limited population highly expressing EpCAM (EpCAM^hi^ cells) by a flow cytometric analysis (**Figure 4A**). Therefore, we isolated EpCAM^hi^ cells as an AEC2 subset by flow cytometric sorting on days 3 and 14 after the BLM instillation (**Figure 4B**) and examined their expression of canonical pro-inflammatory cytokines, chemokines, and growth factors associated with SASP by qRT-PCR (**Figure 4C**). We confirmed that *Cdkn1a* was more highly expressed in BLM-instilled lungs than in saline-instilled lungs on days 3 and 14. Of note, *Cdkn1a* expression levels were >10-fold higher than in saline-instilled lungs on day 3. Although its expression level was decreased on day 14, it was still fourfold higher than in saline-instilled mice. These results are consistent with those shown in **Figure 1F**. We were unable to quantify *Cdkn2a* from 10,000 to 20,000 sorted AEC2, which was the maximum number of cells obtained with high purity from the lungs of a mouse. The canonical SASP-related genes, *Il6* and *Serpine1* encoding plasminogen activator 1 (PAI-1), were expressed at levels that were >5-fold higher than those in control mice on days 3 and 14. Since IL-6 and PAI-1 were previously demonstrated to promote and transmit the senescence of self and neighboring cells (13, 37, 38), these molecules may have contributed to the persistence and augmentation of senescent AEC2 during the progression of BLM-ILD. We also examined inflammatory mediators associated with the migration and activation of monocytes/macrophages because they play pivotal roles in fibrosing ILD (39). The expression levels of *Ccl2*, which chemoattracts circulating monocytes, were 20- and 5-fold higher on days 3 and 14, respectively, than in saline-instilled mice. The expression of *Tnfa*, which activates macrophages, was slightly increased throughout the disease. Fibroblast growth factor genes, including *Tgfb*, *Pdgfa*, and *Pdgfb*, were also examined. High expression levels of *Tgfb* were continuously observed throughout BLM-ILD. On the other hand, significantly high expression levels of *Pdgfa* were detected in the early phase, whereas those in the late phase were similar to those in the control lung. We found no significant increase in *Pdgfb* expression in the early phase and only a slight increase in the late phase. Therefore, AEC2 in BLM-ILD lungs expressed various SASP-related genes, including mediators to promote and transmit senescence and to activate monocytes/macrophages or fibroblasts with temporal differences in the magnitude of each component, suggesting the contribution of senescent AEC2 to the initiation and progression of BLM-ILD in different context-dependent manners.

### Cytokines and chemokines derived from senescent AEC2 contribute to the initiation of lung fibrosis in BLM-ILD

**Figure 5:**
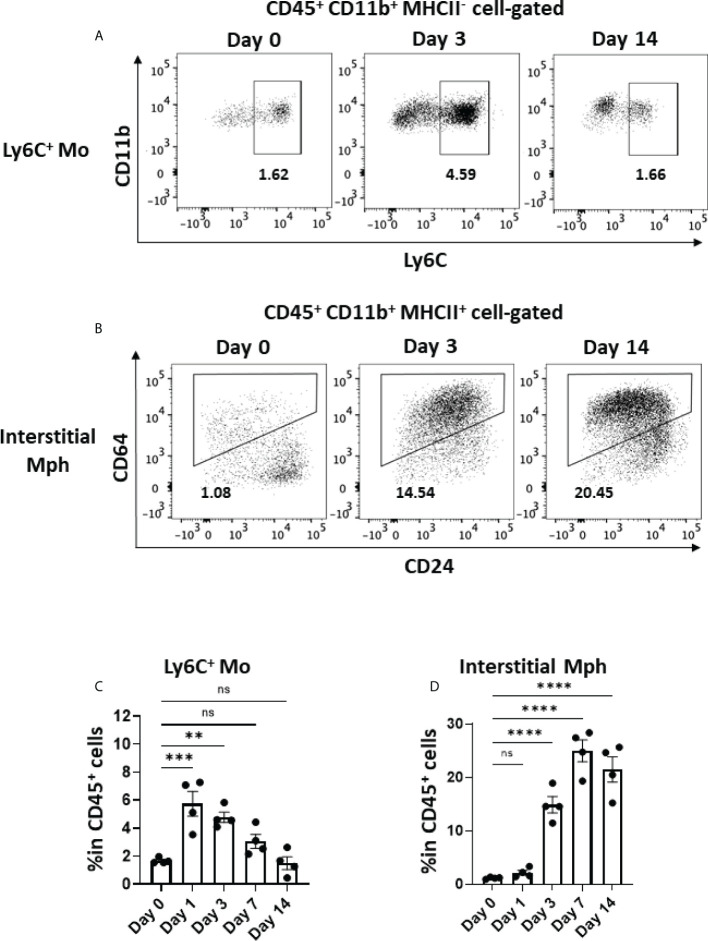
Monocyte migration and accumulated interstitial macrophages in the early phase in BLM-ILD. A lung cell suspension was obtained from mice before and 3 and 14 days after the instillation of BLM and stained with fluorochrome-conjugated antibodies for CD45, CD11b, I-Ab CD24, and CD64. **(A, B)** Representative cytograms showing Ly6C+ monocytes (Mo) **(A)** and interstitial macrophages (Mph) **(B)** on the indicated days. The number indicates the percentage in CD45+ cells. **(C, D)** Frequencies of Ly6C+ monocytes **(C)** and interstitial macrophages **(D)** in CD45+ cells in the lungs from pooled mice. n = 4 per day. Data are presented as the mean ± SEM. Statistics show p-values from a one-way ANOVA with Dunnett’s multiple comparisons test as the post-hoc test comparing values on day 0. ns, not significant. **p < 0.01; ***p < 0.001; ****p < 0.0001.

Activated macrophages play a critical role in the development of lung fibrosis (39). Ly6C^+^ monocytes, which are derived from the circulation and express CCR2, migrate to inflamed tissue through the CCL2–CCR2 interaction (40, 41). As shown in **Figures 5A, C**, Ly6C^+^ monocytes immediately increased in the lungs by approximately threefold within 24 h of the BLM instillation, whereas the frequency of Ly6C^+^ monocytes (CD45^+^CD11b^+^MHCII^-^Ly6C^+^) gradually decreased after day 3. The dynamics of monocyte migration appeared to correlate with the expression of *Ccl2* by AEC2 (**Figures 4C**, **5C**). Furthermore, we found that interstitial macrophages (CD45^+^CD11b^+^MHCII^+^CD64^+^) increased from day 3, which was after monocyte migration (**Figures 5B, D**). Since Ly6C^+^ monocytes differentiate into activated interstitial macrophages that express MHCII under proinflammatory cytokines, including TNFα (42), TNFα produced by senescent AEC2 may have contributed to the increase in interstitial macrophages.

**Figure 6:**
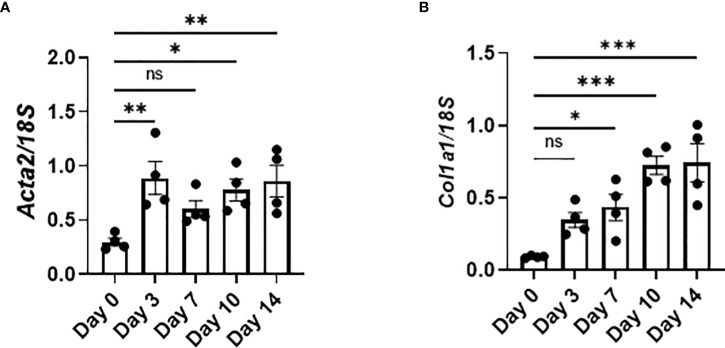
Activated fibroblasts produce collagen from the early phase in BLM-ILD. Lungs were obtained before and 3, 7, 10, and 14 days after the BLM instillation and subjected to qRT-PCR. The expression of Col1a1 **(A)** and Acta2 **(B)** in the lungs on the indicated days. n = 4 per day. Data are presented as the mean ± SEM. Statistics show p-values from a one-way ANOVA with Dunnett’s multiple comparisons test as the post-hoc test comparing values on day 0. ns, not significant. *p < 0.05; **p < 0.01; ***p < 0.001.

The expression of *Acta2*, encoding α-smooth muscle actin, and *Col1a1*, encoding pro-α1 chains of type I collagen, was increased as early as day 3, indicating that the activation of fibroblasts and production of collagen had already been initiated as early as day 3 (**Figures 6A, B**). Therefore, the production of TGFβ and PDGFa by senescent AEC2 in the early phase (**Figure 4C**) may directly promote the activation of fibroblasts and resultant collagen production from the early phase of BLM-ILD.

## Discussion

In this study, we examined the dynamics of the emergence of senescent cells and the characteristics of SASP during the progression of murine BLM-ILD to clarify how cellular senescence and SASP are involved in the pathogenesis of fibrosing ILD. We found that the p21^WAF1/CIP1^ protein was induced in AEC2 as early as day 1 post-BLM instillation, and that p21^WAF1/CIP1^-expressing AEC2 gradually increased and persisted until the later fibrosis phase in BLM-ILD. Its mRNA *Cdkn1a* was transiently and highly expressed in the early phase and then decreased to a certain level that was maintained until the later phase. This initial p21^WAF1/CIP1^-dependent senescence in AEC2 was speculated to be attributed to BLM-induced DNA damage through the activated p53-p21^WAF1/CIP1^ pathway, as previously demonstrated (30). This is consistent with the observation of γ-H2AX expression in a subset of AEC2 in the early phase. AEC2 also expressed another senescent marker, p16^INK4A^, which was expressed as early as day 1 in a small number of AEC2, and increases in p16^INK4A+^ AEC2 numbers were slower and milder than those in p21^WAF1/CIP1+^ AEC2, suggesting that p16^INK4A^-dependent senescence in AEC2 was affected not only by the direct effects of BLM, but also by alterations in the tissue microenvironment caused by inflammatory cell infiltration and the resultant inflammation, apoptosis, and senescence themselves. AEC2 sorted from BLM-ILD lungs in the early phase expressed various inflammatory or profibrotic genes associated with SASP, including *Il6*, *Tnfa*, *Ccl2*, *Serpin1*, *Tgfb*, and *Pdgfa*. We speculated that these inflammatory mediators contributed to the initiation and progression of the disease: IL-6, PAI-1 encoded by *Serpin1*, and TGFβ promoted senescent cells and/or amplified senescence; CCL2 attracted circulating Ly6C^+^ monocytes into the lungs; and infiltrated Ly6C^+^ monocytes were activated by TNFα and differentiated into MHCII-expressing interstitial macrophages. The activation of fibroblasts, as shown by early increases in *Acta2* and *Col1a* immediately upon the BLM instillation, may reflect the production of TGFβ and PDGFa by senescent AEC2 in the early phase.

Several studies have suggested pathological roles for cellular senescence in fibrosing ILD. However, most studies were performed using *in vitro* or *ex vivo* experiments in the chronic phase of fibrosis in human IPF lung samples. Furthermore, only senescent cells and/or SASP in the later phase of fibrosis were investigated in most *in vivo* animal studies, including those involving the depletion of senescent cells using genetically mutated mice or senolytics (31). Therefore, it is important to note that our study demonstrated that p21^WAF1/CIP1^- and p16-dependent senescent cells were involved in both the initiation and progression of chronic fibrosis by clarifying the dynamics of the accumulation of senescent cells dependent on both pathways.

The p21^WAF1/CIP1^ protein was expressed in a large number of AEC2 as early as 1 day after the BLM instillation. DNA damage by BLM activates the p53-p21^WAF1/CIP1^ pathway. If DNA is normally repaired, the replication ability of cells is restored; otherwise, cells undergo senescence or apoptosis (43). We found a divergence in dynamics between the p21^WAF1/CIP1^ protein and its gene expression: with higher levels in the early phase, its gene expression decreased and was maintained at a certain level in the later phase, whereas its protein expression, with lower levels in the early phase, gradually increased as fibrosis progressed. After the onset of DNA damage by BLM and the resultant induction of p21^WAF1/CIP1^, DNA was repaired and p21^WAF1/CIP1^ expression was suppressed in some AEC2. However, DNA damage was not repaired and p21^WAF1/CIP1^ expression persisted in other AEC2, leading to the induction of senescence. Moreover, the p21^WAF1/CIP1^ protein was stabilized post-transcriptionally (44). We speculated that the divergence of the accumulation of the p21^WAF1/CIP1^ protein and the lower expression of *Cdkn1a* in the late stage is attributed to the post-transcriptional regulation of p21^WAF1/CIP1^.

The mechanisms by which senescent AEC2 persisted and increased after the initial stimulus with BLM are unclear. We observed high expression levels of the canonical SASP factors IL-6 and PAI-1 in AEC2 both in the early and late phases of BLM-ILD. Since IL-6 has been reported to promote senescence through the activation of NF-κB in an autocrine manner (16), the BLM-induced senescence of AEC2 may persist by self-produced IL-6. Another canonical SASP factor, PAI-1, was shown to activate the p52-p21^WAF1/CIP1^ pathway and promote the senescence of AEC2 in both autocrine and paracrine manners (37, 38). TGFβ, which was detected in AEC2 from the early phase and throughout the progression of BLM-ILD in this study, has also been reported to induce the senescence of neighboring cells in a paracrine manner (16). Therefore, the persistent expression of IL-6, PAI-1, and TGFβ may amplify senescence across lung tissue by reinforcing senescence, inducing senescence in other cells, and contributing to the accumulation of p21^WAF1/CIP1^-dependent and p16^INK4A^-dependent senescent cells from the early phase throughout BLM-ILD. Therefore, inhibitors of senescent cells and SASP-related mediators have potential as a treatment for ILD.

The present study has several limitations. Although 85% of sorted EpCAM^hi^ cells expressed proSP-C indicating AEC2, SASP-related mediators expressed in EpCAM^hi^ cells may be influenced by those produced by contaminated type 1 AEC. In addition, apoptotic cells produce proinflammatory cytokines, such as IL-1β and TNFα, which overlap with SASP-related cytokines. Therefore, the extent to which apoptotic AEC2 contributed to the expression of some mediators produced by senescent AEC2 in the early phase remains unclear. However, since apoptotic cells do not produce IL-6 (45, 46), most of this cytokine was considered to be derived from senescent AEC2. Another point is that we found differences in the magnitude and components of SASP between the early and late phases of BLM-ILD by examining several genes of SASP-related mediators expressed in AEC2. However, it is important to perform comprehensive analyses of SASP-related mediators expressed in senescent AEC2, such as a single-cell analysis, to understand their roles in diseases and to identify therapeutic targets. Loss-of-function experiments, such as the depletion of p21^WAF1/CIP1^-dependent senescent AEC2 or the inhibition of SASP-related mediators, are required to demonstrate that the accumulation of interstitial macrophages and the initiation of fibrosis at early time points are dependent on the senescence of AEC2.

In summary, we demonstrated that p21^WAF1/CIP1^-dependent and p16^INK4A^-dependent senescent cells emerged from the initiation phase with different dynamics and persisted during the fibrosis phase in murine BLM-ILD. We also showed that SASP-related genes derived from AEC2 were expressed at different magnitudes in the early phase and later fibrosis phase. These results may contribute to a better understanding of the mechanisms involved in cellular senescence as well as useful information for the development of senescent cell- or SASP mediator-targeted therapy.

## Materials and methods

### Mice

Male C57BL/6J mice aged 5–6 weeks were purchased from CLEA Japan Inc. (Tokyo, Japan) and maintained at the animal facility of Toho University. Mice were randomly assigned by a third party and housed in plastic cages (*n* = 3–4 mice per cage) with *ad libitum* water and food under controlled temperature and humidity with a 12-h light/dark cycle. They were housed for 2 to 3 weeks to acclimate them to the environment after shipping. All experimental procedures were conducted in the SPF animal laboratory of Toho University. Animal experiments were performed according to the animal experiment guidelines approved by Toho University Animal Experiment User Committee (Approval numbers: 19-41-432, 20-42-432 and 21-43-432).

### BLM-ILD

Male C57BL/6J mice aged 8–10 weeks were intratracheally instilled with 80 μl of saline containing 3.2 mg/kg body weight of bleomycin sulfate (Nippon Kayaku Co., Ltd., UK) to induce BLM-ILD or 80 μl of saline alone as the control. Mice were anesthetized with an intraperitoneal injection of 0.75 mg/kg of medetomidine (Nippon Zenyaku Kogyo, Fukushima, Japan), 4.0 mg/kg of midazolam (Sandoz, Tokyo, Japan), and 5.0 mg/kg of vetorphale (Meiji Seika Pharma, Tokyo, Japan) before the procedure, and medetomidine was antagonized by a peritoneal injection of 0.75 mg/kg of atipamezole (Nippon Zenyaku Kogyo, Fukushima, Japan) after the procedure. The lungs were dissected under the anesthesia described above before or on day 1, 3, 7, 10, or 14 after the administration of BLM.

### Histological procedure

Lung tissue was fixed with 4% paraformaldehyde and embedded in paraffin. Sections with a thickness of 3 μm were used for HE, MT, and immunohistochemical staining. In the immunohistochemical analysis, sections were deparaffinized and antigen retrieval was performed in antigen retrieval buffer pH 9 (Nichirei Biochemicals Inc., Tokyo, Japan) using a pressure cooker. After the inactivation of endogenous peroxidase activity with 3% H_2_O_2_ for 5 min and subsequent blocking for 30 min, sections were stained with primary antibodies. Information on primary antibodies and horseradish peroxidase-conjugated secondary antibodies are listed with incubation times and dilutions in **Supplementary Table 2**. Histological images of HE or MT staining and immunohistochemical analyses were captured using a BX-63 microscope (Olympus, Tokyo, Japan). Immunofluorescence images were captured using a Nikon ECLIPSE Ti2 Microscope (Nikon, Tokyo, Japan) with the Andor DragonFly Spinning Disk Confocal System (Oxford Instruments, Abington, UK).

### Quantitative histological analysis

An image of the total left lobe area with MT and immunohistochemical staining was divided into grid areas of 240 × 360 µm and 120 × 180 μm, respectively. Pulmonary fibrosis was scored in 20 randomly selected grid areas (240 × 360 µm) by the Ashcroft scale (47) using MT-stained slides, and the average score of each section was calculated. Regarding the quantification of collagen, the MT-stained area and total area of 20 randomly selected grid areas (120 × 180 μm) were measured by ImageJ (NIH, USA) and the average of the percentage of MT areas in the total area was calculated. To quantify p21^WAF1/CIP1+^ AEC2 cells in immunofluorescent images, cells were counted in 20 randomly selected areas (300 × 300 µm).

### Flow cytometric analysis and sorting

Lungs were cut into fine pieces (1 mm^3^) in RPMI1640 containing 2.5 mg/ml collagenase (FUJIFILM Wako Pure Chemical Corporation, Osaka, Japan), 1 mg/ml of dispase II (Roche, Basel Switzerland), and 0.02 mg/ml of DNase I (Millipore, Barrington, USA). The cell suspension was incubated at 37°C for 21 min with pipetting every 7 min and passed through a 70-µm cell strainer. After washing, red blood cells were lysed and Fc receptors were blocked. Cells were stained with fluorescence-conjugated monoclonal antibodies for surface molecules shown in **Supplementary Table 3** and incubated on ice for 15 min. After washing, cells were ready for the flow cytometric analysis of surface molecules. Regarding staining with prosurfactant protein C (proSP-C), cells were further fixed and permeabilized with eBioscience Intracellular Fixation/Permeabilization buffer (Thermo Fisher Scientific, Waltham, USA), and then stained with anti-proSP-C polyclonal Ab (pAb) (Abcam, Cambridge, UK) followed by staining with Alexa Fluor 555-conjugated anti-rabbit pAb (Abcam). The flow cytometric analysis was performed using BD LSRFortessa TM (BD Biosciences, San Jose, USA), and data analyses were conducted using FlowJo software ver. 10.7.1 (BD Biosciences). BD FACS Aria™ III (BD Biosciences) was used for flow cytometric sorting.

### Quantitative RT-PCR

Dissected lungs were soaked in RNA Protect Tissue Reagent (QIAGEN, Hilden, Germany) at 4°C for a few days and subsequently stored at −80°C until the extraction of RNA. Frozen lung tissue immersed in RNA extraction buffer RA1 from NucleoSpin RNA (TAKARA) was homogenized using TissueLyser LT (QIAGEN) and total RNA was extracted following the manufacturer’s protocol. Sorted cells were lysed after adding Trizol LS (Thermo Fisher Scientific) and total RNA was extracted according to the manufacturer’s protocol. Total RNA was reverse-transcribed using the PrimeScript™ RT reagent Kit with the gDNA Eraser (TAKARA) following the manufacturer’s protocol. qRT-PCR was performed on QuantStudio 3 (Thermo Fisher Scientific) using TB Green^®^ *Premix Ex Taq*™ II (TAKARA) and values were normalized to the expression of *18S* ribosomal RNA. Primer sequences are listed in **Supplementary Table 4**.

### Statistical analysis

Statistical analyses were performed using Prism ver. 7.0 software (GraphPad Software, San Diego, USA). A one-way ANOVA was used for multiple-group comparisons. Dunnett’s multiple comparison test was employed as a post-hoc test. An unpaired *t*-test was used for two-group comparisons. *p*-values less than 0.05 were considered to be significant. All data were expressed as the mean ± standard error of the mean (SEM).

## Data availability statement

The raw data supporting the conclusions of this article will be made available by the authors, without undue reservation.

## Ethics statement

The animal study was reviewed and approved by Toho University Animal Care and User Committee.

## Author contributions

ZY, JN, and TN contributed to conception and design of the study. ZY, KM, SM, SY and TM performed experiments. ZY, JN, and KM performed the statistical analysis. ZY, JN, and TN wrote the manuscript. All authors contributed to manuscript revision, read, and approved the submitted version.

## Funding

This work was supported in part by Japan Society for the Promotion of Science (18K07166 and 21K08482); the Private University Research Branding Project from the Ministry of Education, Culture, Sports, Science, and Technology, Japan; the Science Research Promotion Fund from the Promotion and Mutual Aid Corporation for Private Schools of Japan; Takeda Science Foundation; Research Promotion Grant from the Toho University Graduate School of Medicine (17-01, 20-01); and Project Research Grant from the Toho University School of Medicine (20-20).

## Acknowledgments

We thank Kanoh Kondo at the Division of Rheumatology, Department of Internal Medicine, Toho University School of Medicine and Yuichi Tsuchiya at the Department of Biochemistry, Toho University School of Medicine for their technical advice on the immunohistological analysis; Tatsuya Tsukui at the Lung Biology Center, Department of Medicine, University of California for his technical advice on the isolation of mouse lung cells; and Marii Ise at the Department of Molecular Immunology, Toho University for her assistance with the flow cytometric analysis and flow cytometric cell sorting.

## Conflict of interest

The authors declare that the research was conducted in the absence of any commercial or financial relationships that could be construed as a potential conflict of interest.

## Publisher’s note

All claims expressed in this article are solely those of the authors and do not necessarily represent those of their affiliated organizations, or those of the publisher, the editors and the reviewers. Any product that may be evaluated in this article, or claim that may be made by its manufacturer, is not guaranteed or endorsed by the publisher.
