# Corrigendum: Senescence of alveolar epithelial cells impacts initiation and chronic phases of murine fibrosing interstitial lung disease

**DOI:** 10.3389/fimmu.2023.1201209

**Published:** 2023-04-19

**Authors:** Zento Yamada, Junko Nishio, Kaori Motomura, Satoshi Mizutani, Soichi Yamada, Tetuo Mikami, Toshihiro Nanki

**Affiliations:** ^1^Department of Internal Medicine, Toho University Graduate School of Medicine, Tokyo, Japan; ^2^Division of Rheumatology, Department of Internal Medicine, Toho University School of Medicine, Tokyo, Japan; ^3^Department of Immunopathology and Immunoregulation, Toho University School of Medicine, Tokyo, Japan; ^4^Department of Pathology, Toho University School of Medicine, Tokyo, Japan

**Keywords:** interstitial lung disease, p21, p16, senescence-associated secretary phenotype, type 2 alveolar epithelial cell, IL-6, interstitial macrophages

In the published article, there were errors in the approval numbers by Animal Experiment User Committee.

A correction has been made to **Materials and methods**, *Mice*. The numbers were incorrectly written as “Approval number: 19-12-432, 20-12-432 and 21-12-432”. The correct numbers are “Approval numbers: 19-41-432, 20-42-432 and 21-43-432.”

The authors apologize for this error and state that this does not change the scientific conclusions of the article in any way. The original article has been updated.

