# Supplementary material for: Senescence of alveolar epithelial cells impacts initiation and chronic phases of murine fibrosing interstitial lung disease
Source: Front Immunol. 2022 Aug 18;13:935114. doi: 10.3389/fimmu.2022.935114 (PMC9434111; doi:10.3389/fimmu.2022.935114)
Supplement: Supplementary file 1 [file Table_1.docx]

**Supplementary Table 1. Ratio of p21 ^WAF1/CIP1+^ cells to proSP-C^+^ cells in BLM-ILD lung section with double immunofluorescence staining with p21^+^ cells and proSP-C^+^**

|  | Number of  proSP-C^+^ cells | Number of  p21^+^proSP-C^+^ cells | Ratio of p21^+^proSP-C^+^ to proSP-C^+^ cells |
| --- | --- | --- | --- |
| Mean±SD | 20.45±5.54 | 6.6±4.07 | 0.32±0.15 |

proSP-C^+^ or p21 ^WAF1/CIP1+^ (p21^+^) cells were counted in 20 randomly selected areas (300 µm x 300 µm) of BLM-ILD lung section with immunofluorescence staining as shown in Figure 2.
