# Supplementary material for: Senescence of alveolar epithelial cells impacts initiation and chronic phases of murine fibrosing interstitial lung disease
Source: Front Immunol. 2022 Aug 18;13:935114. doi: 10.3389/fimmu.2022.935114 (PMC9434111; doi:10.3389/fimmu.2022.935114)
Supplement: Supplementary file 2 [file Table_2.docx]

**Supplementary Table 2. Antibodies used in this article for immunohistochemistry and immunofluorescence**

| 1^st^ Ab | 2^nd^ Ab | Blocking reagents | Incubation time,  temperature | 1^st^ Ab dilution |
| --- | --- | --- | --- | --- |
| **Immunohistochemistry** | | | | |
| p21  (abcam, ab188224) | ImmPRESS Goat Anti-Rabbit IgG Polymer Kit Peroxidase  (Vector Laboratories, MP-7451) | Protein Block Serum free  (Dako, X0909) | RT, 1hr | 1:1000 |
| p16  (SANTA CRUZ BIOTECHNOLOGY, sc-377412) | Followed by M.O.M. ImmPRESS Polymer Kit Peroxidase  (Vector Laboratories, MP-2400) | | RT, 30min | 1:500 |
| γ-H2AX  (Gene Tex, GTX628789) | Followed by M.O.M. ImmPRESS Polymer Kit Peroxidase  (Vector Laboratories, MP-2400) | | RT, 30min | 1:500 |
| Cleaved caspase-3  (Cell Signaling TECHNOLOGY, #9664) | ImmPRESS Goat Anti-Rabbit IgG Polymer Kit Peroxidase (Vector Laboratories, MP-7451) | Protein Block Serum free  (Dako, X0909) | 4℃, o/n | 1:1000 |
| Ki-67  (BioLegend, 151202) | ImmPRESS  Goat Anti-rat IgG Polymer Kit Peroxidase (Vector Laboratories, MP-7404) | Protein Block Serum free  (Dako, X0909) | RT, 1hr | 1:150 |
| **Immunofluorescence** | | | | |
| proSP-C  (abcam, ab90716) | Alexa Fluor® 555  donkey Anti-Rabbit IgG H&L  (abcam, ab150074) | Donkey Serum 2.5% | RT, 1hr | 1:700 |
| p21  (abcam, ab107099) | Alexa Fluor® 488  donkey Anti-Rat IgG H&L  (abcam, ab150153) | Donkey Serum 2.5% | RT, 1hr | 1:1000 |

RT:　room temperature, o/n: overnight, HRP: horseradish peroxidase
