# Supplementary material for: Senescence of alveolar epithelial cells impacts initiation and chronic phases of murine fibrosing interstitial lung disease
Source: Front Immunol. 2022 Aug 18;13:935114. doi: 10.3389/fimmu.2022.935114 (PMC9434111; doi:10.3389/fimmu.2022.935114)
Supplement: Supplementary file 3 [file Table_3.docx]

**Supplementary Table 3. Fluorescence-conjugated Abs used in flow cytometric analysis and cell sorting**

| Ab | Catalog Number |
| --- | --- |
| PE/Cyanine7 anti-mouse CD31 mAb | Biolegend, 102418 |
| FITC anti-mouse CD45 mAb | Biolegend, 103108 |
| APC anti-mouse CD326 (Ep-CAM) mAb | Biolegend, 118214 |
| Anti-mouse proSP-C pAb | abcam, ab90716 |
| Alexa Fluor® 555 donkey anti-Rabbit IgG H&L | abcam, ab150153 |
| APC/Cyanine7 anti-mouse CD45 mAb | Biolegend, 103115 |
| APC anti-mouse/human CD11b mAb | Biolegend, 101212 |
| PE/Cyanine7 anti-mouse CD11c mAb | Biolegend, 117318 |
| FITC anti-mouse CD24 mAb | Biolegend, 101805 |
| Brilliant Violet 421™ anti-mouse CD64 mAb | Biolegend, 139309 |
| PE anti-mouse I-A/I-E mAb | Biolegend, 107607 |
| PerCP anti-mouse Ly-6C mAb | Biolegend, 128027 |
| Purified anti-mouse CD16/32 mAb | Biolegend, 101302 |
