# Supplementary material for: Senescence of alveolar epithelial cells impacts initiation and chronic phases of murine fibrosing interstitial lung disease
Source: Front Immunol. 2022 Aug 18;13:935114. doi: 10.3389/fimmu.2022.935114 (PMC9434111; doi:10.3389/fimmu.2022.935114)
Supplement: Supplementary file 4 [file Table_4.docx]

**Supplementary Table 4. Primer sequences used for qRT-PCR.**

| Gene | Forward primer | Reverse primer |
| --- | --- | --- |
| *Cdkn1a* | 5’-TCCCGACTCTTGACATTGCT-3’ | 5’-TGCAGAAGGGGAAGTATGGG-3’ |
| *Cdkn2a* | 5’-CTCTGCTCTTGGGATTGGC-3’ | 5’-GTGCGATATTTGCGTTCCG-3’ |
| *Il6* | 5’-ACGATGATGCACTTGCAGAA-3’ | 5’-GTAGCTATGGTACTCCAGAAGA-3’ |
| *Tnfa* | 5’-GGCAGTACTTTGGAGTCAT-3’ | 5’-ACATTCGAGGCTCCAGTGAATT-3’ |
| *Ccl2* | 5’-AACTACAGCTTCTTTGGGACA-3’ | 5’-CATCCACGTGTTGGCTCA-3’ |
| *Serpine1* | 5’-CGTGTCAGCTCGTCTACAG-3’ | 5’-CTATGGTGAAACAGGTGGACT-3’ |
| *Tgfb* | 5’-AGCCCGAAGCGGAACTACTAT-3’ | 5’-TCCACATGTTGCTCCACACT-3’ |
| *Pdgfa* | 5’-GTCCAGGTGAGGTTAGAGG-3’ | 5’-CACGGAGGAGAACAAAGAC-3’ |
| *Pdgfb* | 5’-GTCCAGGTGAGGTTAGAGG-3’ | 5’-ACTTTCGGTGCTTGCCTTTG-3’ |
| *18S* | 5’-GTAACCCGTTGAACCCCATT-3’ | 5’-CCATCCAATCGGTAGTAGCG-3’ |
